# Understanding and advancing research software grant funding models

**DOI:** 10.12688/openreseurope.20210.2

**Published:** 2026-05-11

**Authors:** Daniel S. Katz, Eric A. Jensen, Michelle Barker

**Affiliations:** 1 University of Illinois Urbana-Champaign, Urbana, Illinois, USA; 2 Institute for Methods Innovation, Dublin, Ireland; 3 Research Software Alliance, Portland, OR, USA

**Keywords:** research software, research funding, research policy, digital tools, international research systems, digital infrastructure

## Abstract

Research software funding currently operates across a disconnected landscape of public and private grant-making organizations, leading to inefficiencies for software projects and the broader research community. The lack of coordination forces projects to pursue multiple, often overlapping opportunities, and forces funders to independently evaluate projects and proposals, resulting in duplicated effort and suboptimal resource distribution. By examining existing collaboration models, including centralized and distributed approaches, we highlight how joint decision-making mechanisms could improve sustainability for reusable software resources. An international set of examples illustrates how cross-organization cooperation for research software funding can be structured. Such collaborations can optimize grant disbursement and align priorities. Increased collaboration could allow funders to better address the ongoing maintenance and evolution of research software, lowering barriers that hamper discovery across multiple research domains. Encouraging both bottom-up user-driven and top-down coordination mechanisms ultimately supports more robust, widely accessible research software, improving global research outcomes.

## 1. Introduction

In this paper, we examine the challenges in the way the development and maintenance of research software is currently funded by an array of different public and private organizations. Here, we focus in particular on reusable research software *.* We note that there is typically little coordination between these funding organizations. This leads to inefficiencies for research software projects seeking funding, the funding organizations, and the overall research software field. We also discuss the existing models for cross-organization coordinated funding and models that could reduce inefficiencies and lead to better sustained research software that improves research overall. We recommend that new and existing funders wishing to increase research impact consider coordinated, collaborative approaches to research software resourcing.

## 2. Challenges to funding research software

*Research software* is defined by Gruenpeter *et al.* as “source code files, algorithms, scripts, computational workflows and executables that were created during the research process or for a research purpose,” and distinguished from *software in research*, in turn defined as “software components (e.g., operating systems, libraries, dependencies, packages, scripts, etc.) that are used for research but were not created during or with a clear research intent” ( 2021). They also note that “this differentiation may vary between disciplines,” for example, a compiler could be research software for a computer scientist performing research in compilation or programming languages, but likely would not be for an astrophysicist performing research on gravitational waves. We have chosen to use this term and definition in this paper rather than *scientific software*, as research software is a more inclusive term that also encompasses research fields such as the humanities and engineering.

Research software can be developed by one person/group and used just for their own research, or it can be intended for use by multiple groups. Software can be open source in both cases, since this is a function of licensing or permissions, not intent. In this paper, we focus on the research software that is developed for research use by people other than the developers. To be precise, we could call this *reusable research software,* but for simplicity’s sake, we will refer to it just as *research software* going forward. We do not intend for this to include software that is reused solely for replication of results, which is one reason to make software open source without it being intended for others to use it. Note that Section 3 also contains a description of some of the many previous categorizations of research software.

There is also a challenge in how research software is considered by funders, who are often focused on novelty. The software we are discussing here may be novel, but it is probably better described as infrastructure than research. Most research funders recognize that they need to support and maintain infrastructure to enable the best research to be performed, for instance, instruments (e.g., telescopes, sequencers), data systems (e.g., archives), and platforms (e.g., ocean-going research vessels). However, most do not routinely consider software as part of the research infrastructure that needs support for both development and maintenance. As is widely discussed in the software community, and as captured by Hinsen (2019), software needs ongoing maintenance to continue to work, as it is part of a dynamic environment: it needs to be kept working when the software and hardware used changes; it needs to be kept working correctly when a bug or other problems such as security holes are found; and for it to be useful, it needs to be updated as its research field changes with new applications and new methods.

### 2.1 Funding processes

Having defined what we mean by (reusable) research software in this paper, we now turn to the resources needed to develop and maintain it. These resources can be acquired from the software’s users, as is the case for commercial research software or when a research project contributes funding or personnel to a software project to ensure that it can provide the features or support needed for the research project. The resources can also be acquired from the developers, who may work on the software because they need it for their own research (similar to the previous) because their institution sees it as valuable for future research or reputation. They can also be provided by volunteers, who could be interested in seeing the software succeed for a variety of reasons. Finally, the resources can be acquired from a third party who invests in the research software because they want to support the research it will enable. This is typically the case for research software that receives grants from government, philanthropic, or commercial sources. And this latter case is where we will focus.

The predominant model for grant funding of research software today is one of multiple grant-making organizations and multiple software projects seeking grants. In most cases, the grant-making organizations create calls for proposals with some focus, perhaps in terms of the discipline(s) that the software should impact, the type of software work that will be supported (development of new software, maintenance of existing software, conversion of existing private software into a community tool, community growth, increased diversity, etc.) The process by which an organization decides what areas to support varies widely, and inputs can be either internal or external, including the organization’s management, external advisory bodies, or higher-level stakeholders (e.g., a government ministry). In this paper, we assume this decision process has already happened, and we focus on the implementation of the funding program.

A large number of research grant-funding programs exist, but only a small number focus on research software. Many of the latter are captured and shared by the Research Software Alliance (ReSA) in its public database of current and past research software funding opportunities ( Research Software Alliance, 2025). Section 3 of this paper discusses some examples.

In this environment, it is up to research software projects that need support to be aware of all relevant grant opportunities and to write proposals to each, while being aware of possible policies against the submission of the same work to multiple organizations and against receiving multiple grants for the same work.

The grant-making organization then determines what to fund, typically using peer review. This review process can either provide inputs on the value of the proposals that the organization can use to determine which software to support, or the peer-review process can make the decisions for the organization.

In this model, there is no coordination between the decision-making processes of each opportunity as provided by different organizations, which can lead to some projects being awarded multiple grants and others none, without full regard for the need for the software work. And because very few software projects are independent of all others, this can lead to a failure to support software projects on which other software projects, and in turn, research projects, are dependent.

A call for proposals is not the only model for grant-making organizations. Some organizations also work in a more discretionary and less open manner, where staff members find projects to which they want to consider making grants, and then work in private to plan and provide such grants. From the point of view of a software project seeking support, this has both positive and negative aspects. The positive part is that they may not need to be aware of funding opportunities, as the opportunity comes to them. Negative parts include that this may support well-known, well-publicized, and well-connected projects (or their leaders) rather than those with an equal need for support but not the same reputation or interpersonal connections. This also does not resolve the problem of the overall funding opportunities across funders not being matched to the needs across projects.

### 2.2 Funding challenges

Research software projects typically begin under a grant. Because research grants are of fixed duration, the project needs to find new resources to continue to develop and/or maintain the software when the grant ends. This becomes an ongoing process for most software projects, where the resources needed are typically a mix of grant funding for people to do work, volunteers and collaborators, and other resources, and the work to be done includes software work such as development and maintenance, documentation, and testing, along with community work, such as user support, community development, etc. A typical small- to medium-sized project moves between times when resources are available because of a grant and work continues smoothly, and times when these resources are not available when project work falls to what volunteers can provide while they are also seeking new resources. Larger projects with staff dedicated to sustainability (including fundraising) can stay in the smoother periods more often. This is captured in workaround software project lifecycles, e.g., Yehudi *et al.* (2025), which proposed a lifecycle model that includes the concept of challenge events, such as a resource shortfall, and how these can affect the project.

In this paper, however, we focus on the challenges seen by funding organizations. Some issues are specific to funding research software, particularly understanding what is needed (required functions/capabilities) by research communities. Challenging questions include:

• Which software tools are most frequently used by scientists in any given field?
• How does the use of open source compare to proprietary software in any given field?
• Are emerging new tools replacing legacy ones?
• What is the prevalent programming language in any given field?
• Which software tools should be part of a student’s computational curriculum?
• Which software projects should funders prioritize as critical infrastructure for science? ( CZI, 2024)

These kinds of priority-setting and implementation challenges have also been documented in an international survey of research software funders ( Jensen & Katz, 2025a).

Beyond this, multiple funding programs or organizations that want to collaborate, perhaps with multiple countries or organizations pooling resources to address common challenges, often encounter barriers that hinder collaboration. Based on our experiences as research funders and in discussions with funders while running the ReSA Funders Forum and its three conferences, these include:

• Alignment challenges. Divergent priorities due to different strategic goals or geographic focus areas, and timeline mismatches on granting cycles and decision-making processes.
• Risk aversion. Loss of control or reputational concerns related to collaborating on large-scale programs.
• Relationship and trust barriers. Lacking the established relationships needed to connect with other funders, and trust deficits as building the trust necessary for effective collaboration takes time.
• Operational complexity. Governance issues when establishing shared models for decision-making and accountability, and measurement and reporting expectations for reporting outcomes, along with differing requirements on proposals, and different policies for project budgets and project operations.
• Legal and compliance issues. Regulatory complexity due to differing compliance requirements across jurisdictions, and lack of legal frameworks to participate in pooled funding initiatives, where funds contributed by multiple funders are distributed through a centralized process, not by each funder individually
• Resource constraints. Limited administrative capacity and financial limitations, such as a lack of discretionary funds needed to participate in pooled funding.

Some funders have chosen to collaborate to address these challenges, and some models of successful collaboration are presented in Section 4.

## 3. Grant-funding programs for research software

The Research Software Alliance (ReSA) maintains a public database of current and past research software-specific funding opportunities (2025), and here we provide examples of some grant-funding programs, taken in part from that database. Table 1 shares examples across some of these categories to illustrate the types of research funding that currently exist. There are a number of different aspects to these programs, such as funder type, disciplinary focus, stage of software supported, funding mechanism, and duration of funding. An explanation of these and other relevant aspects is given below.

**Table 1.** Illustrative examples of research software funding categories.

Funding Program	Funder	Funder Type	Disciplinary Focus	Stage of Research Software Supported	Funding Mechanism
Building Sustainable Software Tools for Open Science	National Institutes of Health (NIH)	National Government - USA	Biomedical research	Enhancement; infrastructure	Standalone grants
Concept Grants	Sage	Industry	Social science	Prototype; enhancement	Standalone grants
Digital Technology Development Awards (Climate-Sensitive Infectious Disease Modelling)	Wellcome Trust	Philanthropic	Health	Prototype; enhancement	Standalone grants
Research Software - Quality Assured and Reusable	German Research Foundation (DFG)	National Government - Germany	Interdisciplinary	Enhancement; infrastructure	Standalone grants
Support for Open-Source Tools, Frameworks, and Libraries	NASA	National Government - USA	Space science	Infrastructure	Standalone grants
Supplement for Open-Source Science	NASA	National Government - USA	Space and earth science	Prototype; enhancement	Supplement to existing award

An example of a national and disciplinary-focused program that supports existing research software is NIH’s Building Sustainable Software Tools for Open Science (OD-24-010). This program supports enhancing the sustainability and impact of research software tools by enabling the use of best practices and design principles in software development and by leveraging continuing advances in computing. This call is also aimed at facilitating the creation of partnerships between developers and users of software and tools, and promoting FAIR (Findable, Accessible, Interoperable, Reusable) ( Chue Hong *et al.*, 2022) practices for research software to maximize research value.

While rarer, there are also industry research software funding programs. For example, the academic publisher Sage’s Concept Grants program funds innovative products and tools aimed at enhancing social science education and research. One class of awards is for Doing Research: Developing advanced research software tailored for academic research in the social sciences ( Sage Research Methods Community, 2024).

The Digital Technology Development Awards (Climate-Sensitive Infectious Disease Modelling) program, run by the UK-based Wellcome Trust philanthropy, is designed to fund the development of digital tools that will improve climate-sensitive infectious disease modelling and support their maintenance later on. This funding is aimed at supporting the delivery of open-source digital technology that improves the impact of research relevant to this theme of climate-sensitive infectious disease modelling.

An example of national cross-disciplinary funding is the German Research Foundation’s (DFG’s) program, Research Software: Quality Assured and Reusable. This is an interdisciplinary call for proposals from German organizations that pursues three sub-goals, usability and impact, quality assurance, and further development, which together seek to raise the maturity level of research software so that it can also be used by researchers other than those developing it, and to simplify its further development.

An example of a national and disciplinary-focused program that supports existing research software is NASA’s Support for Open-Source Tools, Frameworks, and Libraries (OSTFL, ROSES-24 F.7). This supports the improvement and sustainment of existing software that has significantly impacted the NASA Science Mission Directorate (SMD) science community. The program element seeks to support two types of awards. 1) Foundational awards: cooperative agreements for up to five years for open-source tools, frameworks, and libraries that have a significant impact on two or more divisions of the SMD and 2) Sustainment awards: grants or cooperative agreements of up to three years in duration for open-source tools, frameworks, and libraries that have significant impact in one or more divisions of the SMD.

An example of a nationally and disciplinary-focused program that is only available to existing grantees is NASA’s Supplement for Open-Source Science opportunity (ROSES-24 F.8) NASA supports adding an open science component to an existing “parent” ROSES (NASA’s overall Research Opportunities in Space and Earth Sciences funding program) award. The goal of this program element is to increase the accessibility, inclusivity, and reproducibility of the science from the parent award, to contribute back to the open-science communities relevant to the parent award, and/or provide cloud credits to further support or expand the parent award. This could include taking software developed in the project supported by the parent award and further developing it for additional usage.

### 3.1 Funder type

The funder type (and the funding model) category differentiates between the kinds of various organizations that financially support research software projects. A key funding source is governmental funding, usually national. Government agencies provide substantial resources to advance research and development nationally. Governmental funding is often driven either top-down by political priorities and strategic initiatives or bottom-up by research community requests and expert decision-making. In some countries, similar funding structures exist at the sub-national level, for example, in Brazil with the São Paulo Research Foundation (FAPESP) and the Fundação de Amparo à Pesquisa do Estado do Rio de Janeiro (FAPERJ) in the states of São Paulo and Rio de Janeiro respectively. More unusual are supranational funding structures, such as those administered by the European Commission on behalf of European Union Member States and Associated Countries.

Another source is philanthropic funding. Philanthropic funders such as the Chan Zuckerberg Initiative (CZI) may focus on specific areas of interest or innovative approaches that align with their mission and values. They often aim to complement or fill gaps left by governmental funding programs, and sometimes collaborate directly with governmental funders. A third category is industry funding, provided by companies.

### 3.2 Disciplinary focus

This category addresses the scope of research fields targeted by funding programs.

Some programs have a *specific discipline/field* focus, aiming to advance software development within particular scientific areas such as space sciences or biomedical research. For example, NASA’s programs are tailored to support software that benefits space and Earth sciences, while NIH and CZI focus on research software for biomedical science. A limited disciplinary range can limit options for coordination between funders.

Conversely, *inter/multidisciplinary* programs are open to multiple disciplines or encourage cross-disciplinary collaboration.

It is also relevant to consider the stages of software supported.

This category considers the development phase of the research software projects that funding programs support. Here, we use a three-stage classification:

1. *Prototype development* (Stage 1) refers to creating early-stage software, where initial concepts are transformed into functional prototypes.
2. *Enhancement of existing software* (Stage 2) involves improving or adding new features to already developed software. This could include optimizing performance, expanding functionality, or adapting the software for additional use cases. Likewise, *maintenance and sustainment* (Stage 2) focus on the ongoing support required to keep software operational, secure, and up-to-date. This includes activities like bug fixes, updates, and user support.
3. *(Research) Infrastructure development* (Stage 3) pertains to building foundational systems or platforms that support other research software applications. Funding for infrastructure is designed to create robust environments and structures upon which other tools and applications can be developed and executed.

There have been many previous categorizations of research software, such as work by Hinsen (2019) on the layers of software above the operating system, from 1, non-scientific infrastructure such as gcc and Python, and R, to 2, scientific infrastructure such as BLAS, HDF5, SciPy, and the tidyverse, to 3, domain-specific tools such as GROMACS and MMTK, to 4, project-specific code such as scripts, notebooks, and workflows. Hasselbring *et al.* (2025) offer a multi-axis approach, where one axis is “role in research”, which includes proof-of-concept software and research infrastructure software as categories. Maturity is another axis for Hasselbring *et al.*, 2025, with categories of research data processes, novel methods and models, and accepted methods and models. This maturity axis was originally proposed for the Australian Research Data Commons (ARDC) by Honeyman and Treloar (2021), who highlight that different activities are needed at each level: increased sharing for research data processes, application of best practices to novel methods and models, and increased maintenance for accepted methods and models.

An alternative model was developed by the German Aerospace organization, Deutsches Zentrum für Luft- und Raumfahrt (DLR) ( Schlauch *et al., *2018). It includes 4 classes, from 0) personal use and small scope and not planned for distribution outside of the local organization, to 1) possible for non-developers to use and open to extension/development by external people, to 2) having a defined development process and a plan for long-term development and maintenance, to 3) critical software with product characteristics. The European Virtual Institute for Research Software Excellence (EVERSE) has released a Research Software Quality Kit (RSQkit) ( https://everse.software/RSQKit) which includes a three-tier view ( EVERSE, 2024). This is based on the ARDC model, consisting of analysis code, prototype tools, and research software infrastructure, though this also roughly corresponds to DLR’s class 0, 1–2, and 2–3, respectively.

Our proposed categorization above follows from this previous work, most closely following ARDC’s model, but with slight changes to better fit this more general context.

### 3.3 Funding mechanism

The funding mechanism category describes how funds are allocated to research software projects. A *supplement to an existing award* is additional funding to augment current projects. This mechanism allows researchers to expand the scope of their work, integrate new components, or address unforeseen challenges without securing a completely new grant. A supplement to a research grant that supported software development for that research might be used to generalize the software to support the research of others.

In contrast, *standalone grants* are independent funding opportunities that do not need to be tied to existing research projects. These grants enable researchers to initiate new projects or pursue innovative ideas independently of their current work. Within this category are (a) standalone *grants for research that include research software projects* and (b) dedicated funding specifically for research software per se.

## 4. Existing coordination of funding organizations

A range of models for funding programs designed to address shared international priorities collaboratively exists. These models typically involve funding organizations either pooling their resources to be centrally distributed or agreeing to work together under specific conditions, with funders only providing resources to their local teams selected for funding through a shared process. Here, we present some examples of such funding models, highlighting the potential to draw inspiration from such established structures.

Few examples exist of philanthropic funders coordinating their efforts and running shared programs specifically focusing on research software. We focus here on four examples that illustrate both philanthropic and government funder collaborations, and pooled funding that is administered either by a single centralized process or through separate but coordinated processes, as shown in Table 2.

**Table 2.** Illustrative examples of research software funding collaborations.

Funders	Program	Funder Type	Administration
CZI, Kavli Foundation, Wellcome Trust	Essential Open Source Software for Science (EOSS)	Philanthropic	Separate, coordinated processes
Gordon and Betty Moore Foundation, Footprint Coalition, Schmidt Futures	Low-Cost Tools for Science	Philanthropic +	Centralized
CHIST-ERA consortium members	Open and Re-usable Research Data and Software	Government	Separate, coordinated
European Commission	Horizon Europe Framework Programme (HORIZON): Digital and emerging technologies for competitiveness and fit for the Green Deal: Fundamentals of Software Engineering	Government	Centralized

Another example of multi-funder coordination that provides a different model, albeit one not yet seen with a focus on research software, is the Belmont Forum, which coordinates both national government and philanthropic funders.

### 4.1 CZI, Kavli Foundation, and Wellcome Trust: EOSS

These three international philanthropic funders collaborated on the sixth cycle of the EOSS program in 2024. The program supports open-source software projects that are essential to biomedical research, specifically the software maintenance, growth, development, and community engagement for these critical tools. It seeks projects from all over the world that have previously demonstrated impact, currently show potential for continued improvement, and are expected to deliver added value to the biomedical research community through the proposed activities.

While CZI was the sole funder in the first five rounds of the EOSS opportunity, the Kavli Foundation and Wellcome Trust joined CZI in the sixth. CZI managed the program and the peer-review process. After that, each funder chose the software they wanted to support, and the funders discussed this, leading to more coordinated funding decisions. This improves the situation for the software projects funded, compared to them having to apply independently to these three funders, followed by the funders running separate evaluation processes and making independent decisions. While it reduces the work for software projects and leads to more coordinated decisions, it does not include many other funders, so the projects cannot rely on only this program ( Hertweck *et al., *2024).

### 4.2 Gordon and Betty Moore Foundation, Footprint Coalition, Schmidt Futures: Low-cost tools for science

This 2023 program supported projects that aim to build and/or test new low-cost tools for science. Low-cost science tools can increase access to scientific experimentation and inquiry for individuals and organizations that may not have the resources to purchase expensive laboratory equipment and supplies. This can help close the gap between those with access to science resources and those without access. Low-cost science tools can also help to encourage innovation by providing individuals and organizations with the resources they need to develop and test new ideas and theories. This can lead to discoveries and advancements in scientific knowledge. This small, fast grant program aimed to get new ideas off the ground quickly, backing eligible projects up to USD$10,000. Funds were distributed on a first-come, first-served basis ( Experiment, 2023).

### 4.3 CHIST-ERA: Open and re-usable research data and software

CHIST-ERA is a consortium of research funding organisations in Europe and beyond supporting use-inspired basic research in Information and Communication Technologies (ICT) or at the interface between ICT and other domains. This collaborative funding structure is limited to the European Research Area. It has core funding from the European Commission and brings national funders together to create a transnational call for proposals. Each year, CHIST-ERA’s participating funding organizations identify emerging scientific domains that enable European researchers to undertake high-risk, high-impact initiatives to advance knowledge. In pursuit of this goal, they issue transnational calls for proposals focused on two priority ICT topics. These calls are formulated and published within six months of selecting topics, drawing on the research community’s expertise and a strategic long-term vision to maintain their relevance in rapidly evolving research fields.

In 2023, one of CHIST-ERA’s funding calls was focused on: Open and Re-usable Research Data and Software. This call tackles the challenge of open research data and software from the perspective of their possible reuse. While there was a total funding pool of 6 million euros, each partner in the successful research projects is funded separately by their respective national/regional research funding organization. This is an example of the model where each funder provided resources to the research teams that are within their typical geographical scope; each research project has to include a minimum of 3 partners requesting funding from participating funding organizations from at least 3 of the following countries: Belgium, Brazil, Czech Republic, France, Latvia, Lithuania, Luxembourg, Poland, Romania, Slovakia, Switzerland, Turkey, United Kingdom ( CHIST-ERA, 2024).

### 4.4 European Commission - Horizon Europe Framework Programme (HORIZON), Digital and emerging technologies for competitiveness and fit for the Green Deal: Fundamentals of Software Engineering

This call aims to progress the state of the art in responsible software engineering methods, tools, and best practices leveraging, among others, novel AI and data technologies to accelerate the development and maintenance of software, including for multi-architecture systems, addressing in particular efficient and agile modelling, verification, and validation, as well as vulnerability assessment and mitigation. Projects are expected to demonstrate their developments in at least three industrial or societal use cases. Implementing responsible software engineering, the use-cases should address functional and non-functional requirements and principles like optimising energy usage, reducing the environmental footprint, security-by-design, and data protection ( European Commission, 2022).

### 4.5 Belmont Forum

The Belmont Forum is an international partnership that mobilizes funding of environmental change research. Since its formation in 2009, the Forum has successfully led 22 calls for proposals, supporting 181 projects in over 90 countries. Each of the Belmont Forum’s funding programs comprises an ad hoc coalition gathered to address a shared focus called a Collaborative Research Action. The Forum works to bring prospective partners into the scoping phase at the earliest juncture so they can co-produce the developing Collaborative Research Action agenda. Yet this process remains fluid enough for organizations, whether they offer monetary or in-kind support, to join at any point, reflecting the realities of collaborative engagement between research funders that operate on different timescales. Each participant’s contributions, core thematic interests, eligibility parameters, and primary program contacts are documented in a publicly available document linked to the Collaborative Research Action funding call. Awards are made through coordinated funding from individual organizations, that is, the funding is not administered directly by the Belmont Forum, but by the collaborating organizations to the research projects within their remit (which is often geographic but can also be international) ( Belmont Forum, 2017).

## 5. Options to democratize funding decision-making and coordination

We propose three main categories of decision-making for research software funding: (1) funder-driven; (2) user-driven; (3) developer-driven ( Table 3).

**Table 3.** Locus of decision-making in research software funding.

Decision- making Locus	Definition
Funder-driven	Research software funders take into account strategic priorities, perceived research community needs and other factors to determine the guidelines and priorities for funding. Allocation decision-making may be implemented through formal peer review processes, expert panels, or funder representatives.
User-driven	Researchers’ needs are expressed through their usage of specific software, and this drives funding allocation decisions.
Developer-driven	Software developers analyze the needs of the research community, usage patterns, software needs, and other variables, and collectively use this to determine where to allocate resources.

The models we have discussed so far are all funder-driven, though with differences in how the decisions are actually made, and in possible coordination mechanisms between funders. Typically, public (e.g., government) research funders make decisions based on peer review that evaluates potential projects, and this is also used by some philanthropic funders and industry. Some funders make decisions based on their own knowledge of needs, perhaps informed by internal and/or external feedback on one or more projects selected for potential funding.

In the other two categories, funders still provide resources, but the community determines how to allocate them. This could be done at two different levels: software projects and researchers/users. The funder-driven category can be viewed as top-down, while the software developer-driven category can be viewed as bottom-up, with the user-driven model in the middle.

At the software project level, the Sustainable Research Software Institute proposal ( Watson *et al.*, 2023) proposed a system where similar software projects talk about what they need to improve their sustainability. Then representatives of those groups work together to make an overall allocation of funding that matches what funders provide. The Sustainable Research Software Institute proposal was a predecessor to what is now the CORSA project ( https://corsa.center) within the US Department of Energy-funded Consortium for the Advancement of Scientific Software (CASS, https://cass.community/). CASS partially implements this with software projects that were formerly funded by DOE’s Exascale Computing Project (ECP, https://www.exascaleproject.org), which have been grouped into CASS projects that share some common discipline, intent, or practices. Each CASS project has an internal process where the software projects within it propose the sustainability funding they need, and the CASS project collectively decides the funding split. The funding given to each CASS project is currently determined by the funder (DOE), but an extension of the current system could allow the CASS projects to decide on this split collectively.

At the user level, funding could be distributed to projects based on their usage by researchers funded by (or of importance to) particular funders. A mechanism such as Drips (Decentralized Research Infrastructure for Pursuing Sustainability, https://www.drips.network/app) could track the usage of software by such research projects, and report to the funders of the projects, who could then use this information to distribute funding to the software developers. The funders would determine how this funding would be allocated, for example, based on the number of their researchers who use the software, the relative importance of the researchers to the funders’ goals, or another factor.

No matter what decision-making mechanism is used (or combined), coordination between funders is generally beneficial, as at the least, shared knowledge between funders can lead to better decisions, where funders can make decisions with awareness and in response to those of other funders (e.g., funder A is aware that funder B is supporting project X and so chooses to support project Y instead of also supporting project X). Active shared decision making can be even more efficient, where multiple funders coordinate their decisions (e.g., funders A and B collectively decide that projects X and Y should be funded, and how funding should be most effectively split and distributed).

## 6. Conclusion

Research software underpins much of contemporary scholarship, yet the funding pillars supporting it are not well-coordinated and aligned to maximize efficiency and impact. Multiple agencies and organizations—governmental, philanthropic, and industry—provide grants with limited coordination, often creating duplicative efforts for both funders and recipients. A central issue lies in the mismatch between software projects’ ongoing resource needs and time-limited grant cycles, leading to funding gaps, maintenance bottlenecks, and gaps in support for critical tools. For example, a recent study of international research software funders describes how funding strategies are evolving in response to sustainability and coordination pressures ( Jensen & Katz, 2025c).

New frameworks for more efficient, coordinated funding, drawing on cross-organization communication and infrastructure sharing, are emerging in the international research funding landscape. Existing collaborative structures like CHIST-ERA, the Belmont Forum, and philanthropic partnerships from CZI illustrate how shared goals can unify diverse funders and reduce inefficiencies. Pooled resources, co-funded calls, and community-driven allocation can help identify software tools with broad applicability and critical dependencies across research fields. In addition to the models provided above, other useful inputs include the Amsterdam Declaration on Funding Research Software Sustainability (ADORE.software), which aims to raise awareness of the role of funding practice in the sustainability of research software and to improve that practice. The Declaration includes a set of recommendations and an accompanying ADORE.software Toolkit, which have gained some traction with research software funders ( Jensen & Katz, 2025a, 2025b).

Several options for funding models and procedures have emerged to distribute resources and make decisions between competing options and priorities. Traditional peer review remains prevalent as an input for funder-driven decisions. Other approaches place decision-making power with users (viz., via usage-based funding allocations) or software developers (e.g., through collective negotiations of funding pools). A mix of processes may be effective to align funding decisions with both real-world needs and strategic considerations.

Groups such as the ReSA-led Research Software Funders Forum and the Global Research Council (GRC) Multilateral Engagement Working Group are also a focus for useful discussions. Bringing philanthropic and governmental funders into collaborative initiatives can help research software projects gain consistent support to evolve from prototype to sustainable infrastructure. Such alignment reduces duplication and strengthens the software ecosystem via more efficient resource allocation, ensuring that tools vital for advancing the frontiers of knowledge are robust, widely accessible, and continuously improved.

Finally, as stated by reviewers of the first version of this paper and audience members where we have spoken, the incentive system in research performing institutions remains an underlying issue. Multiple groups are working to address this, including Coalition for Advancing Research Assessment (CoARA) in its Agreement on Reforming Research Assessment published in July 2022 ( Arentoft *et al.*, 2022), as well as the ReSA/RDA Policies in Research Organisations for Research Software (PRO4RS) working group ( Barker *et al.*, 2025).

### Ethics and consent

Ethical approval and consent were not required.

## Disclaimer

The views expressed in this article are those of the authors. Publication in Open Research Europe does not imply endorsement by the Alfred P. Sloan Foundation or the European Commission.

## Data Availability

All materials underlying the article are contained within the text.
