# Supplementary material for: Understanding and advancing research software grant funding models
Source: Open Res Eur. 2026 May 11;5:199. Originally published 2025 Jul 25. [Version 2] doi: 10.12688/openreseurope.20210.2 (PMC12743209; doi:10.12688/openreseurope.20210.2)
Supplement: Peer review history

# Reviewer report: Reviewer response for version 2

**DOI:** 10.21956/openreseurope.25049.r74018

**Published:** 2026-05-12

**Authors:** Jukka Ruohonen

**Affiliations:** 1 University of Southern Denmark, Sønderborg, Denmark

I had eight concerns. All were adequately addressed in the revision. I further acknowledge that the open letter format caused some confusion for the previous review.

Where applicable, are recommendations and next steps explained clearly for others to follow? (Please consider whether others in the research community would be able to implement guidelines or recommendations and/or constructively engage in the debate)

Partly

Does the article adequately reference differing views and opinions?

Partly

Are all factual statements correct, and are statements and arguments made adequately supported by citations?

Yes

Is the rationale for the Open Letter provided in sufficient detail? (Please consider whether existing challenges in the field are outlined clearly and whether the purpose of the letter is explained)

Yes

Is the Open Letter written in accessible language? (Please consider whether all subject-specific terms, concepts and abbreviations are explained)

Yes

Reviewer Expertise:

Computer science, software engineering, cyber security, technology policy, European Union, among other things

I confirm that I have read this submission and believe that I have an appropriate level of expertise to confirm that it is of an acceptable scientific standard.

# Reviewer report: Reviewer response for version 2

**DOI:** 10.21956/openreseurope.25049.r74017

**Published:** 2026-05-12

**Authors:** Ludo Waltman

**Affiliations:** 1 Leiden University, Leiden, The Netherlands

No further comments.

Where applicable, are recommendations and next steps explained clearly for others to follow? (Please consider whether others in the research community would be able to implement guidelines or recommendations and/or constructively engage in the debate)

Yes

Does the article adequately reference differing views and opinions?

Yes

Are all factual statements correct, and are statements and arguments made adequately supported by citations?

Yes

Is the rationale for the Open Letter provided in sufficient detail? (Please consider whether existing challenges in the field are outlined clearly and whether the purpose of the letter is explained)

Yes

Is the Open Letter written in accessible language? (Please consider whether all subject-specific terms, concepts and abbreviations are explained)

Yes

Reviewer Expertise:

open science; scholarly communication; peer review; research assessment; scientometrics

I confirm that I have read this submission and believe that I have an appropriate level of expertise to confirm that it is of an acceptable scientific standard.

# Reviewer report: Reviewer response for version 1

**DOI:** 10.21956/openreseurope.21867.r64286

**Published:** 2025-12-26

**Authors:** Ludo Waltman

**Affiliations:** 1 Leiden University, Leiden, The Netherlands

In this ‘open letter’ the authors argue there is a lack of coordination in funding for research software. According to the authors, this lack of coordination leads to inefficiencies, in particular “duplicated effort and suboptimal resource distribution”. The authors argue that coordination between funders of research software needs to be strengthened, and they provide examples of ways in which such coordination can be organized.

 I don’t consider myself to be an expert on the specific issue of funding for research software. However, on the basis of my broader expertise on issues related to research funding and open science, I found the paper to be clear and informative. Based on the information available on the Open Research Europe platform, my understanding is that an ‘open letter’ should discuss “policies relevant to a broad research community”, that it “should usually represent the views of an eligible project or group of projects”, and that it “should not include new data and analysis”. To me the paper appears to meet the criteria for an ‘open letter’. I don’t have any specific suggestions for improvements.

Where applicable, are recommendations and next steps explained clearly for others to follow? (Please consider whether others in the research community would be able to implement guidelines or recommendations and/or constructively engage in the debate)

Yes

Does the article adequately reference differing views and opinions?

Yes

Are all factual statements correct, and are statements and arguments made adequately supported by citations?

Yes

Is the rationale for the Open Letter provided in sufficient detail? (Please consider whether existing challenges in the field are outlined clearly and whether the purpose of the letter is explained)

Yes

Is the Open Letter written in accessible language? (Please consider whether all subject-specific terms, concepts and abbreviations are explained)

Yes

Reviewer Expertise:

open science; scholarly communication; peer review; research assessment; scientometrics

I confirm that I have read this submission and believe that I have an appropriate level of expertise to confirm that it is of an acceptable scientific standard.

# Response

**Published:** 2026-02-10

**Authors:** Eric Jensen

**Affiliations:** Institute for Methods Innovation, Ireland

Dear Ludo Waltman,   Thank you for taking the time to review this and for providing your positive feedback. We hope you will view the changes we have made in response to other reviewers as making the paper stronger.

# Response

**Published:** 2026-05-03

**Authors:** Eric Jensen

**Affiliations:** Institute for Methods Innovation, Ireland

Dear Ludo Waltman,   Thank you for taking the time to review this and for providing your positive feedback. We hope you will view the changes we have made in response to other reviewers as making the paper stronger.

# Reviewer report: Reviewer response for version 1

**DOI:** 10.21956/openreseurope.21867.r64287

**Published:** 2025-12-18

**Authors:** Mahirah Kamaludin

**Affiliations:** 1 Universiti Malaysia Terengganu, Kuala Terengganu, Terengganu, Malaysia

This Open Letter provides a clear and timely overview of the fragmented landscape of research software funding and makes a persuasive case that better coordination among funders could improve sustainability and efficiency. The article successfully maps existing funder types, funding mechanisms, and collaborative models, using international examples that help readers understand how joint decision-making and pooled approaches can work in practice. The manuscript is accessible, logically structured, and well suited to its intended policy-oriented audience. It is particularly strong as a primer for both software developers and funders who are unfamiliar with the current funding ecosystem, and it appropriately highlights the growing role of international coordination efforts in strengthening research software as critical infrastructure.

 However, to improve the manuscript, the authors should strengthen the evidential basis of their main claims and better align the abstract, body, and conclusion. Several key statements about inefficiencies, project dependency, and the benefits of coordination require clearer empirical support or more explicit framing as informed observations rather than established facts. The definitions of “research software” and the proposed software stages would benefit from tighter consistency and brief justification of how these categories were constructed. The conclusion should be more specific about how proposed coordination and community-driven models would operate in practice, and should acknowledge structural issues beyond funding, such as academic incentive systems and the high long-term costs of software maintenance. Adding short clarifying sentences on these points would significantly strengthen the manuscript’s coherence and policy relevance.

Where applicable, are recommendations and next steps explained clearly for others to follow? (Please consider whether others in the research community would be able to implement guidelines or recommendations and/or constructively engage in the debate)

Partly

Does the article adequately reference differing views and opinions?

Partly

Are all factual statements correct, and are statements and arguments made adequately supported by citations?

Yes

Is the rationale for the Open Letter provided in sufficient detail? (Please consider whether existing challenges in the field are outlined clearly and whether the purpose of the letter is explained)

Yes

Is the Open Letter written in accessible language? (Please consider whether all subject-specific terms, concepts and abbreviations are explained)

Yes

Reviewer Expertise:

-

I confirm that I have read this submission and believe that I have an appropriate level of expertise to confirm that it is of an acceptable scientific standard, however I have significant reservations, as outlined above.

# Response

**Published:** 2026-02-10

**Authors:** Eric Jensen

**Affiliations:** Institute for Methods Innovation, Ireland

Response to Reviewer 4   Dear Mahirah Kamaludin,   Thank you for taking the time to review this and for providing your feedback.   Here, we respond to your points that bring up issues, including a brief summary of each point: *Original point: … to improve the manuscript, the authors should strengthen the evidential basis of their main claims and better align the abstract, body, and conclusion. Several key statements about inefficiencies, project dependency, and the benefits of coordination require clearer empirical support or more explicit framing as informed observations rather than established facts. *   This open letter (which is not a research paper) is based in part on our experiences (as funders and in discussions with funders while running events for research funders such as the ReSA Funders Forum and its three conferences). We've added text in Section 2.2 to make this clearer, which we feel is the primary place that contains our own opinions, unsupported by external references. We've also added a few words at the start of Section 5 to make clear that this section is a proposed model. *Original point: The definitions of “research software” and the proposed software stages would benefit from tighter consistency and brief justification of how these categories were constructed.*   The definition of research software is external and based on a previous community discussion, so we view it as more or less fixed at this time. We have added a sentence at the end of the software stages part of Section 3 to better contextualize our proposed stages. *Original point: The conclusion should be more specific about how proposed coordination and community-driven models would operate in practice, and should acknowledge structural issues beyond funding, such as academic incentive systems and the high long-term costs of software maintenance. *     We don't feel that we are ready to make specific recommendations, but rather, that this is the point where more discussion is needed, which we hope to have contributed to. Ideally, this discussion will lead to further research and piloting, and then recommendations from ourselves and others. We have added a paragraph at the end of the conclusions that discusses the academic incentive system as a concern.   Additionally, we updated the Yehudi et al. reference from a poster to a paper that was published after our original paper was submitted.

# Response

**Published:** 2026-05-03

**Authors:** Eric Jensen

**Affiliations:** Institute for Methods Innovation, Ireland

**AUTHOR RESPONSE**

 Dear Mahirah Kamaludin,   Thank you for taking the time to review this and for providing your feedback.   Here, we respond to your points that bring up issues, including a brief summary of each point:  

 Original reviewer point: … to improve the manuscript, the authors should strengthen the evidential basis of their main claims and better align the abstract, body, and conclusion. Several key statements about inefficiencies, project dependency, and the benefits of coordination require clearer empirical support or more explicit framing as informed observations rather than established facts. 

 AUTHOR RESPONSE: This open letter (which is not a research paper) is based in part on our experiences (as funders and in discussions with funders while running events for research funders such as the ReSA Funders Forum and its three conferences). We've added text in Section 2.2 to make this clearer, which we feel is the primary place that contains our own opinions, unsupported by external references. We've also added a few words at the start of Section 5 to make clear that this section is a proposed model.  

 Original point: The definitions of “research software” and the proposed software stages would benefit from tighter consistency and brief justification of how these categories were constructed.

 AUTHOR RESPONSE: The definition of research software is external and based on a previous community discussion, so we view it as more or less fixed at this time. We have added a sentence at the end of the software stages part of Section 3 to better contextualize our proposed stages.  

 Original point: The conclusion should be more specific about how proposed coordination and community-driven models would operate in practice, and should acknowledge structural issues beyond funding, such as academic incentive systems and the high long-term costs of software maintenance. 

 AUTHOR RESPONSE: We don't feel that we are ready to make specific recommendations, but rather, that this is the point where more discussion is needed, which we hope to have contributed to. Ideally, this discussion will lead to further research and piloting, and then recommendations from ourselves and others. We have added a paragraph at the end of the conclusions that discusses the academic incentive system as a concern.   Additionally, we updated the Yehudi et al. reference from a poster to a paper that was published after our original paper was submitted

# Reviewer report: Reviewer response for version 1

**DOI:** 10.21956/openreseurope.21867.r64289

**Published:** 2025-12-18

**Authors:** Filomena Canterino

**Affiliations:** 1 Polytechnic University of Milan, Milan, Italy

This Open Letter addresses the fragmented landscape of research software funding and advocates for stronger coordination among European and international research funders. The authors argue that uncoordinated funding mechanisms create inefficiencies for software developers, funders, and the broader research ecosystem. The letter provides an overview of existing grant-funding programs across governmental, philanthropic, and industry sectors to illustrate the variety of current approaches. It also presents examples of existing coordination models among funding organizations, highlighting both centralised and distributed methods for collaborative decision-making. Finally, the authors propose three options to democratize funding decisions, moving beyond traditional funder-driven models to include user-driven and developer-driven mechanisms as alternative pathways for allocating resources more equitably and effectively.

 I believe this piece is relevant and timely, and it offers useful insights for the research community. 

 With the intent of providing constructive feedback for improvement, below I provide detailed comments about the evaluation criteria expressed above

 1. The rationale is clearly stated and appropriate for the Open Letter format. The authors articulate the challenges arising from fragmented and uncoordinated research software funding and convincingly explain why improved coordination is needed. The motivation is grounded in concrete challenges faced by software maintainers, funders, and the broader research system.

 2. The letter presents a strong case for coordinated funding. A suggestion could be to expand a bit alternative viewpoints. For example, authors could include explicit discussion of potential reservations regarding coordination (e.g., autonomy of national funders, risk of over-centralisation, disciplinary differences), or they could reference existing debates from research infrastructure policy literature about collaborative governance.

 3. The descriptions of funding programs and collaborative mechanisms are generally accurate, but I suggest to support general claims about inefficiencies, funding gaps, or the scarcity of research-software-specific programs with explicit references (in the text) to studies, reports, or community surveys.

 4. The letter is readable and clear for audiences familiar with research software and research infrastructure, but accessibility can be further improved. Here two specific points of attention: (i) define specialised terms (e.g., FAIR principles, pooled funding, research infrastructure), (ii) Ensure all acronyms are defined at first use (e.g., CASS, ECP, DRIPS); (iii) break up long paragraphs in Sections 3 and 4 to enhance readability.

 5. The letter offers valuable insights into possible coordination mechanisms but would benefit from more explicit, actionable recommendations. For example, authors could: discuss more explicit a recommendations section, outlining concrete steps funders could implement (e.g., shared review frameworks, exploratory coordinated calls, data-sharing mechanisms for software usage); provide examples of how European funders or research organisations could engage with the proposed user-driven and developer-driven models.

Where applicable, are recommendations and next steps explained clearly for others to follow? (Please consider whether others in the research community would be able to implement guidelines or recommendations and/or constructively engage in the debate)

Partly

Does the article adequately reference differing views and opinions?

Yes

Are all factual statements correct, and are statements and arguments made adequately supported by citations?

Partly

Is the rationale for the Open Letter provided in sufficient detail? (Please consider whether existing challenges in the field are outlined clearly and whether the purpose of the letter is explained)

Yes

Is the Open Letter written in accessible language? (Please consider whether all subject-specific terms, concepts and abbreviations are explained)

Partly

Reviewer Expertise:

Collaborative Research; research ecosystems

I confirm that I have read this submission and believe that I have an appropriate level of expertise to confirm that it is of an acceptable scientific standard, however I have significant reservations, as outlined above.

# Response

**Published:** 2026-02-10

**Authors:** Eric Jensen

**Affiliations:** Institute for Methods Innovation, Ireland

Dear Filomena Canterino,  

 Thank you for taking the time to review this and for providing your feedback.  

 Here, we respond to your points that bring up issues, including a brief summary of each point:  

 Original point 2: The letter presents a strong case for coordinated funding. A suggestion could be to expand a bit alternative viewpoints. For example, authors could include explicit discussion of potential reservations regarding coordination (e.g., autonomy of national funders, risk of over-centralisation, disciplinary differences), or they could reference existing debates from research infrastructure policy literature about collaborative governance.   We believe that the second set of points in Section 2.2 (the challenges) sufficiently discuss these reservations.  

 Original point 3: The descriptions of funding programs and collaborative mechanisms are generally accurate, but I suggest to support general claims about inefficiencies, funding gaps, or the scarcity of research-software-specific programs with explicit references (in the text) to studies, reports, or community surveys.   We agree with this suggestion in theory and thought about it when writing the original draft, but were unable to find such references.  

 Original point 4: Here two specific points of attention: (i) define specialised terms (e.g., FAIR principles, pooled funding, research infrastructure), (ii) Ensure all acronyms are defined at first use (e.g., CASS, ECP, DRIPS); (iii) break up long paragraphs in Sections 3 and 4 to enhance readability.   We have made changes in the text in response, though we note that some of the identified acronyms were already defined when first used.  

 Original point 5: The letter offers valuable insights into possible coordination mechanisms but would benefit from more explicit, actionable recommendations. …   We don't feel that we are ready to make such recommendations, but rather, that this is the point where more discussion is needed between a range of relevant parties, which we hope to contribute to. Ideally, this discussion will lead to future research and then recommendations from ourselves and others. In the meantime, we have added references to relevant prior empirical research with funders.   Additionally, we updated the Yehudi et al. reference from a poster to a paper that was published after our original paper was submitted.

# Response

**Published:** 2026-05-03

**Authors:** Eric Jensen

**Affiliations:** Institute for Methods Innovation, Ireland

**AUTHOR RESPONSE**

  Dear Filomena Canterino, Thank you for taking the time to review this and for providing your feedback. Here, we respond to your points that bring up issues, including a brief summary of each point:

 *Original point 2: The letter presents a strong case for coordinated funding. A suggestion could be to expand a bit alternative viewpoints. For example, authors could include explicit discussion of potential reservations regarding coordination (e.g., autonomy of national funders, risk of over-centralisation, disciplinary differences), or they could reference existing debates from research infrastructure policy literature about collaborative governance.*

 AUTHOR RESPONSE: We believe that the second set of points in Section 2.2 (the challenges) sufficiently discuss these reservations.

 *Original point 3: The descriptions of funding programs and collaborative mechanisms are generally accurate, but I suggest to support general claims about inefficiencies, funding gaps, or the scarcity of research-software-specific programs with explicit references (in the text) to studies, reports, or community surveys.*

 AUTHOR RESPONSE: We agree with this suggestion in theory and thought about it when writing the original draft, but were unable to find such references.

 *Original point 4: Here two specific points of attention: (i) define specialised terms (e.g., FAIR principles, pooled funding, research infrastructure), (ii) Ensure all acronyms are defined at first use (e.g., CASS, ECP, DRIPS); (iii) break up long paragraphs in Sections 3 and 4 to enhance readability.*

 AUTHOR RESPONSE: We have made changes in the text in response, though we note that some of the identified acronyms were already defined when first used.

 *Original point 5: The letter offers valuable insights into possible coordination mechanisms but would benefit from more explicit, actionable recommendations. …*

 AUTHOR RESPONSE: We don't feel that we are ready to make such recommendations, but rather, that this is the point where more discussion is needed between a range of relevant parties, which we hope to contribute to. Ideally, this discussion will lead to future research and then recommendations from ourselves and others. In the meantime, we have added references to relevant prior empirical research with funders. Additionally, we updated the Yehudi et al. reference from a poster to a paper that was published after our original paper was submitted.

# Reviewer report: Reviewer response for version 1

**DOI:** 10.21956/openreseurope.21867.r57224

**Published:** 2025-08-22

**Authors:** Katherine Skinner

**Affiliations:** 1 invest in open infrastructure, USA, USA

I admit not fully understanding the Open Letter format and its norms; I've tried to rely on the description provided ( https://open-research-europe.ec.europa.eu/for-authors/article-guidelines/social-sciences/preparing-an-open-letter/) in order to fairly evaluate this writing. The Open Letter format is intended for, "discussing policies relevant to a broad research community, presenting guidelines or white papers, or announcing new initiatives. An Open Letter should usually represent the views of an eligible project or group of projects; publication does not imply endorsement by the European Commission. An Open Letter should not include new data and analysis..." 

 Based on this description, I see this Open Letter as a limited review of current public/private funder practices specifically in the funding area of research software.

 I see value in the general topic of the Open Letter, which provides a brief, provocative, position around research software public and private funding opportunities, stating that there is a need for more coordination and shared systems for grant makers to cure what it diagnoses as "inefficiencies for research software projects seeking funding, the funding organizations, and the overall research software field.". The letter provides little by way of evidence of concrete benefit that would come from such coordination, though; it also provides little evidence of explicit harm that results from the lack of coordination.

 The authors' hypothesis and methods were not provided as far as I could tell (which may be common for an Open Letter format).

 The letter makes a few steps that I question below, but overall, it is a good review of some of the practices, challenges, and barriers in a particular type of grant-making. It is a solid primer for prospective research software grantees without a clear idea of how these funding opportunities work or where they are; it also provides a good overview for funders in this discipline/topical/functional area on some of the practices and known issues in private/public grant-making as they are experienced from a grantee's point of view.

 Other than a few specific questions I raise below, this seems to be a wholly appropriate Open Letter, with clear recommendations and requests that the target audience should understand.

 A few specific things that I'll call attention to:

 1. The Abstract ambitiously claims that "Encouraging both bottom-up user-driven and top-down coordination mechanisms ultimately supports more robust, widely accessible research software, improving global research outcomes. *" *This statement is unsubstantiated (and perhaps not actually explored) by the Open Letter. Supporting it would require different evidence than this Open Letter provides.

 2. The Open Letter defines "research software" and briefly describes a set of funder models and programs. It then outlines "Funding challenges" in 2.2, making a number of claims that are not substantiated, including "Research software projects typically begin under a grant...the project needs to find new resources to continue to develop and/or maintain the software when the grant ends." While this is true of certain types of software projects (and the authors do specify that they are focused on research software that is developed with the intent that others beyond the developer(s) use the software), there are many other ways that research software projects begin, including as part of student or faculty work that is voluntary or supported by their institutional home or as part of industry work that is voluntary or supported by a commercial entity. Not all of these projects need new resources beyond the developers' volunteerism to continue, though many do. 

 3. The Funding challenges listed in this section are all believable, but again not well substantiated or given clear context. I wonder if the authors could provide clearer signaling via stating these as challenges they themselves have seen or experienced, challenges that others have shared with them, or challenges documented in the scholarly record? Again that may be asking for more than is appropriate in an Open Letter.

 4. I wanted more details on the categories in the ReSA public database of current/past research software-specific funding opportunities (a terrific resource), specifically how these categories were determined and who provides these designations. Of particular interest, the "stage of software supported" gives a three-stage classification that feels academically driven and might leave out a lot of projects. The specificity of the letter's focus on research software developed with the intention of external use (by someone other than the developer) might give enough context to make these three stages make sense, but I can imagine a lot of exceptions to these stages that could be funded, including use of the software by someone other than the developer.

 5. Similarly, I would have loved to see more about how the three main categories of decision-making for research software funding were constructed. In my experience, there are a lot of blended models that do not fit easily into these three categories or their combination; knowing more about who created them and how they fit their purpose within the database would be helpful context. 

 6. I found the arguments about coordination and governance interesting, but underdeveloped. The conclusion didn't match the abstract or introduction well. Claims were made in the conclusion that were often not clearly substantiated, either by the examples provided in the letter or through citations. Again, this may be a product of the "Open Letter" format.

Where applicable, are recommendations and next steps explained clearly for others to follow? (Please consider whether others in the research community would be able to implement guidelines or recommendations and/or constructively engage in the debate)

Yes

Does the article adequately reference differing views and opinions?

Partly

Are all factual statements correct, and are statements and arguments made adequately supported by citations?

Partly

Is the rationale for the Open Letter provided in sufficient detail? (Please consider whether existing challenges in the field are outlined clearly and whether the purpose of the letter is explained)

Yes

Is the Open Letter written in accessible language? (Please consider whether all subject-specific terms, concepts and abbreviations are explained)

Yes

Reviewer Expertise:

Open Infrastructure funding models, Sociology of Business, Sociology of Culture, Business lifecycles, Nonprofit management, etc.

I confirm that I have read this submission and believe that I have an appropriate level of expertise to confirm that it is of an acceptable scientific standard, however I have significant reservations, as outlined above.

# Response

**Published:** 2026-02-10

**Authors:** Eric Jensen

**Affiliations:** Institute for Methods Innovation, Ireland

Dear Katherine Skinner,  

 Thank you for taking the time to review this and for providing your feedback.  

 Here, we respond to your general and numbered points, including a brief summary of each point:  

 A. General point: The letter provides little by way of evidence of concrete benefit that would come from such coordination, though; it also provides little evidence of explicit harm that results from the lack of coordination.   We agree with this; however, we do not have such evidence to prove the point, but we feel the issue will be accepted by almost all developers who have sought grants, and we also believe that many funders have discussed this. Both our experiences (as funders and in discussions with funders while running in ReSA Funders Forum and its three conferences) and the few coordinated programs that exist are evidence that those funders agree with this point. Also see response point 3 below.  

 1. Original point: the open letter claims that "Encouraging both bottom-up user-driven and top-down coordination mechanisms ultimately supports more robust, widely accessible research software, improving global research outcomes." This statement is unsubstantiated (and perhaps not actually explored) by the Open Letter. Supporting it would require different evidence than this Open Letter provides.   We agree, but also believe that the point of the open letter is to propose this idea, not to demonstrate or prove it, which could be the aim of a future research paper.  

 2. Original point: some reusable research software does not need grant funding and can be supported by volunteers.   We agree with this point, but our experience indicates that this is somewhat rare for the type of software we are talking about. However, we've added a sentence in the first paragraph of Section 2.1 to make this situation explicit.  

 3. Original point: The Funding challenges listed in this section are all believable, but again not well substantiated or given clear context. I wonder if the authors could provide clearer signaling via stating these as challenges they themselves have seen or experienced, challenges that others have shared with them, or challenges documented in the scholarly record?   In response to this reasonable comment, we've added text before and after the second list in Section 2.2, and also point out that the first list is referenced by a funder (CZI, 2024), which is the source of those questions.  

 4. Original point: I wanted more details on the categories in the ReSA public database of current/past research software-specific funding opportunities (a terrific resource), specifically how these categories were determined and who provides these designations. Of particular interest, the "stage of software supported" gives a three-stage classification that feels academically driven and might leave out a lot of projects. …   The categories were determined by the authors based on our own analysis of the database contents. While we would welcome further work by others in this area, which might include different categories, we believe these categories are a reasonable representation, and where we are aware of other work (such as the stages of research software), we've attempted to explain our choices in that context.  

 5. Original point: Similarly, I would have loved to see more about how the three main categories of decision-making for research software funding were constructed. In my experience, there are a lot of blended models that do not fit easily into these three categories or their combination; knowing more about who created them and how they fit their purpose within the database would be helpful context.   We agree that blended models are possible, but our point was more to say that funder-directed (top-down) decisions were not the only option and to suggest alternatives. In our experience as funders and based on the funders we have worked with, other options are unlikely to have been considered and do not appear to have been used, except in rare experiments.  

 6. Original point: I found the arguments about coordination and governance interesting, but underdeveloped. The conclusion didn't match the abstract or introduction well. Claims were made in the conclusion that were often not clearly substantiated, either by the examples provided in the letter or through citations. Again, this may be a product of the "Open Letter" format.   As suggested by the reviewer, this is an open letter, not an evidence-based research paper, intended to open discussion that could lead to future empirical research. However, we have added references to prior relevant empirical research with research software funders.   Additionally, we updated the Yehudi et al. reference from a poster to a paper that was published after our original paper was submitted.

# Response

**Published:** 2026-05-03

**Authors:** Eric Jensen

**Affiliations:** Institute for Methods Innovation, Ireland

**AUTHOR RESPONSE**

 Dear Katherine Skinner, Thank you for taking the time to review this and for providing your feedback. Here, we respond to your general and numbered points, including a brief summary of each point:

 A. General reviewer point: The letter provides little by way of evidence of concrete benefit that would come from such coordination, though; it also provides little evidence of explicit harm that results from the lack of coordination. A

 UTHOR RESPONSE:  We agree with this; however, we do not have such evidence to prove the point, but we feel the issue will be accepted by almost all developers who have sought grants, and we also believe that many funders have discussed this. Both our experiences (as funders and in discussions with funders while running in ReSA Funders Forum and its three conferences) and the few coordinated programs that exist are evidence that those funders agree with this point. Also see response point 3 below.  

 1. Original point: the open letter claims that "Encouraging both bottom-up user-driven and top-down coordination mechanisms ultimately supports more robust, widely accessible research software, improving global research outcomes." This statement is unsubstantiated (and perhaps not actually explored) by the Open Letter. Supporting it would require different evidence than this Open Letter provides.

 AUTHOR RESPONSE:  We agree, but also believe that the point of the open letter is to propose this idea, not to demonstrate or prove it, which could be the aim of a future research paper.

 AUTHOR RESPONSE: 

 2. Original point: some reusable research software does not need grant funding and can be supported by volunteers.

 AUTHOR RESPONSE:  We agree with this point, but our experience indicates that this is somewhat rare for the type of software we are talking about. However, we've added a sentence in the first paragraph of Section 2.1 to make this situation explicit.  

 3. Original point: The Funding challenges listed in this section are all believable, but again not well substantiated or given clear context. I wonder if the authors could provide clearer signaling via stating these as challenges they themselves have seen or experienced, challenges that others have shared with them, or challenges documented in the scholarly record?

 AUTHOR RESPONSE:  In response to this reasonable comment, we've added text before and after the second list in Section 2.2, and also point out that the first list is referenced by a funder (CZI, 2024), which is the source of those questions.  

 4. Original point: I wanted more details on the categories in the ReSA public database of current/past research software-specific funding opportunities (a terrific resource), specifically how these categories were determined and who provides these designations. Of particular interest, the "stage of software supported" gives a three-stage classification that feels academically driven and might leave out a lot of projects. …

 AUTHOR RESPONSE:  The categories were determined by the authors based on our own analysis of the database contents. While we would welcome further work by others in this area, which might include different categories, we believe these categories are a reasonable representation, and where we are aware of other work (such as the stages of research software), we've attempted to explain our choices in that context.  

 5. Original point: Similarly, I would have loved to see more about how the three main categories of decision-making for research software funding were constructed. In my experience, there are a lot of blended models that do not fit easily into these three categories or their combination; knowing more about who created them and how they fit their purpose within the database would be helpful context.

 AUTHOR RESPONSE:  We agree that blended models are possible, but our point was more to say that funder-directed (top-down) decisions were not the only option and to suggest alternatives. In our experience as funders and based on the funders we have worked with, other options are unlikely to have been considered and do not appear to have been used, except in rare experiments.  

 6. Original point: I found the arguments about coordination and governance interesting, but underdeveloped. The conclusion didn't match the abstract or introduction well. Claims were made in the conclusion that were often not clearly substantiated, either by the examples provided in the letter or through citations. Again, this may be a product of the "Open Letter" format.

 AUTHOR RESPONSE:  As suggested by the reviewer, this is an open letter, not an evidence-based research paper, intended to open discussion that could lead to future empirical research. However, we have added references to prior relevant empirical research with research software funders. *Additionally, we updated the Yehudi et al. reference from a poster to a paper that was published after our original paper was submitted.*

# Reviewer report: Reviewer response for version 1

**DOI:** 10.21956/openreseurope.21867.r57225

**Published:** 2025-08-14

**Authors:** Jukka Ruohonen

**Affiliations:** 1 University of Southern Denmark, Sønderborg, Denmark

I applaud the authors for addressing the topic of funding for research software. The topic is important and extends well-beyond research software. Nevertheless, regarding criticism, my concerns are about a lack of research questions, the definitions used and framing done, and the general motivation. Thus, in no particular order of importance:

 1. The questions presented in Section 2.2 are relevant but do not match exactly what is presented.  Which one of these is examined in the paper specifically? Regarding the subsequent list of challenges, I suppose alignment is what the conclusion is about.  If so, it should be made explicit in the concluding section. A clarification is needed also whether or not the other challenges are still present too.

 2. The motivation should include a paragraph about software maintenance itself, which is costly.  Therefore, most software projects have a fixed life cycle.  It might take months, years, or decades, but most software is not meant to live forever. In many cases, once a software is released, it is not really an asset but a cost, as also concepts such as technical debt demonstrate. For instance, security updates are needed, and also for research software [1].  Against these backdrops, I would like to see a stronger argument somewhere, perhaps in the concluding section, about why software maintenance itself has supposedly not been a target for funding (?).  While grant-based funding may certainly be a factor, maybe it is also because software maintenance fits poorly into the whole paradigm of research, development, and innovation?

 3. Related to the previous point and regarding the software stages presented in Section 3, it would be relevant to have a cross-tabulation of these and the funding bodies.

 4. The definition for research software in the beginning of Section 2 does not match well the software stages described in Section 3.  By a reasonably well-grounded hypothesis, the overwhelming majority of research software, as per the definition provided, is about software needed to compute results for a single paper or for multiple papers in a research project. Practically all papers dealing with quantitative data nowadays contain such software.  Yet, such software is not really even about prototypes (Stage 1).  Nor is such software even meant to be maintainable.  On one hand, the situation is unfortunate because it affects reproducibility and direct replications of existing results.  On the other hand, it is also unreasonable to demand scientists to maintain such software.  For better or worse, also scientific papers have a fixed live cycle in this regard.

 5.  Although SciPy (but not NumPy, PyTorch, and others) is mentioned, I am surprised to see no mention of R, which is the other *de facto* programming language for statistical computing in particular.

 6. Regarding the stages, the categorization presented seems to lack the fundamental infrastructures used to develop, maintain, and distribute research software. Among these are PyPI for Python and CRAN for R. Furthermore, these language-specific repositories are closely tied to "native" package managers used in Linux distributions and other operating systems.  Thus, all in all, the infrastructures are not only about building foundational systems.  This point correlates with well known security issues currently plaguing open source software repositories.

 7.  Regarding the conclusion, I agree that better coordination and governance are needed.  However, I would also like to see a sharper reflection about what the authors specifically have in mind regarding improvements.  Although community-driven allocation is mentioned, how exactly would it work?  There are also risks involved in using community-based metrics (cf.  [2]), as some funding bodies for non-scientific open source software have done in the past. Given the grant-based funding and prioritization difficulties, perhaps it would make sense to have also larger software foundations involved in strategic consultations or even funding decisions?  After all, they usually have a good bird's-eye view on their respective ecosystems.

 8. Finally, I would also like to see a remark in the concluding section that the issues with research software are not only about funding but also related to the incentive systems in academia.  Strengthening the creation *and* maintenance of research software in academic reward systems would also help at tackling the issues.  The recent Agreement on Reforming Research Assessment (CoARA) has already taken steps toward this direction. 

 [1] https://arxiv.org/pdf/2203.06502

 [2] https://arxiv.org/abs/2412.13459

Where applicable, are recommendations and next steps explained clearly for others to follow? (Please consider whether others in the research community would be able to implement guidelines or recommendations and/or constructively engage in the debate)

Partly

Does the article adequately reference differing views and opinions?

Partly

Are all factual statements correct, and are statements and arguments made adequately supported by citations?

Yes

Is the rationale for the Open Letter provided in sufficient detail? (Please consider whether existing challenges in the field are outlined clearly and whether the purpose of the letter is explained)

Yes

Is the Open Letter written in accessible language? (Please consider whether all subject-specific terms, concepts and abbreviations are explained)

Yes

Reviewer Expertise:

Computer science, software engineering, cyber security, technology policy, European Union, among other things

I confirm that I have read this submission and believe that I have an appropriate level of expertise to confirm that it is of an acceptable scientific standard, however I have significant reservations, as outlined above.

# Response

**Published:** 2026-02-10

**Authors:** Eric Jensen

**Affiliations:** Institute for Methods Innovation, Ireland

Dear Jukka Ruohonen,

 Thank you for taking the time to review this and for providing your feedback. Here, we respond to your numbered points below (labelled 'original point'), including a brief summary of each point:

*1. Original point: questions in Sec. 2.2 are not aligned with the paper's content.*

 This is not a research paper, and these are not research questions we are addressing. Rather, they are motivation and context for the discussion of existing programs in Sec. 3, the existing coordination of programs in Sec. 4, and the discussion and suggestions in Sec. 5.  

*2. Original point: motivation should include a paragraph about software maintenance itself, which is costly.  …  I would like to see a stronger argument somewhere, perhaps in the concluding section, about why software maintenance itself has supposedly not been a target for funding.*  

 We've added a new paragraph in the introduction to Section 2 in response to this good point.   

*3. Original point: Related to the previous point and regarding the software stages presented in Section 3, it would be relevant to have a cross-tabulation of these and the funding bodies.*  

 We believe Table 1 does this sufficiently for this paper.  

*4. Original point: Our focus isn't on the majority of research software, that which is needed to compute results for a single paper or for multiple papers in a research project.*  

 We agree with you that we aren't focusing on the single use software you describe as most common, but on software that is intended for re-use. We believe we have made this point in the second paragraph of Section 2.  

*5. Original point: R is not mentioned with SciPy.*  

 We've added a mention of R and of tidyverse, but we can never exhaustively mention all software or all programming languages.  

*6. Original point: package management systems are not discussed in the stages as part of infrastructure.*  

 While we agree that package managers are a key component of software and programming today, they are not research software, which is where we are focusing. To clarify this, we've added the word research just before our list of stages.  

*7a. Original point: The review would like more about what we specifically have in mind regarding improvements.*  

 We don't feel this is needed in this open letter, but agree that it would be useful in a more detailed article, perhaps to be written later.  

*7b. Original point: It would make sense to have larger software foundations involved in strategic consultations or even funding decisions.*  

 We agree, and have slightly altered the third paragraph of the conclusions to try to make this more clear.  

*8. Original point: The issues with research software are not only about funding but also related to the incentive systems in academia.  Strengthening the creation and maintenance of research software in academic reward systems would also help at tackling the issues.*

  We agree with this point, and have added a new final paragraph in the conclusions.  Additionally, we updated the Yehudi et al. reference from a poster to a paper that was published after our original paper was submitted.

# Response

**Published:** 2026-05-03

**Authors:** Eric Jensen

**Affiliations:** Institute for Methods Innovation, Ireland

AUTHOR RESPONSE

 Dear Jukka Ruohonen, Thank you for taking the time to review this and for providing your feedback. Here, we respond to your numbered points, including a brief summary of each point:

 1. Original point: questions in Sec. 2.2 are not aligned with the paper's content. This is not a research paper, and these are not research questions we are addressing. Rather, they are motivation and context for the discussion of existing programs in Sec. 3, the existing coordination of programs in Sec. 4, and the discussion and suggestions in Sec. 5\.

 2. Original point: motivation should include a paragraph about software maintenance itself, which is costly. … I would like to see a stronger argument somewhere, perhaps in the concluding section, about why software maintenance itself has supposedly not been a target for funding. We've added a new paragraph in the introduction to Section 2 in response to this good point.

 3. Original point: Related to the previous point and regarding the software stages presented in Section 3, it would be relevant to have a cross-tabulation of these and the funding bodies. We believe Table 1 does this sufficiently for this paper.

 4. Original point: Our focus isn't on the majority of research software, that which is needed to compute results for a single paper or for multiple papers in a research project. We agree with you that we aren't focusing on the single use software you describe as most common, but on software that is intended for re-use. We believe we have made this point in the second paragraph of Section 2\.

 5. Original point: R is not mentioned with SciPy. We've added a mention of R and of tidyverse, but we can never exhaustively mention all software or all programming languages.

 6. Original point: package management systems are not discussed in the stages as part of infrastructure. While we agree that package managers are a key component of software and programming today, they are not research software, which is where we are focusing. To clarify this, we've added the word research just before our list of stages.

 7a. Original point: The review would like more about what we specifically have in mind regarding improvements. We don't feel this is needed in this open letter, but agree that it would be useful in a more detailed article, perhaps to be written later.

 7b. Original point: It would make sense to have larger software foundations involved in strategic consultations or even funding decisions. We agree, and have slightly altered the third paragraph of the conclusions to try to make this more clear.

 8. Original point: The issues with research software are not only about funding but also related to the incentive systems in academia. Strengthening the creation and maintenance of research software in academic reward systems would also help at tackling the issues. We agree with this point, and have added a new final paragraph in the conclusions. Additionally, we updated the Yehudi et al. reference from a poster to a paper that was published after our original paper was submitted.
